# With the Pousse Café

**Published:** 1892-04

**Authors:** 


With the Pousse Café. Being a collection of post-parandial verses, by Wm. Tod Helmuth, M. D. Philadelphia (1011 Arch St.) Bœricke & Tafel, 1892. Price, $1.50, net.

This attractive little volume is a collection of after-dinner poems, some of which were read and others given in response to toasts at medical banquets. All are bright and witty, and if amusing to laymen, will be much more so to those of the medical profession, and will recall to many pleasant memories of those reunions where they were given. The paper is elegant, the printing tasty, and the margin all that is to be desired. We agree in all the sentiments, save the Doctor's theory of trusting more the blade than the dose. *Similia similibus curantar*, well and wisely applied, is more powerful than the "sword."

